# Using the Herschel–Bulkley Consistency Index to Characterise Complex Biopolymer Systems—The Effect of Screening

**DOI:** 10.3390/polym16192822

**Published:** 2024-10-06

**Authors:** Anand Raja, Philipp K. Wilfert, Stephen J. Picken

**Affiliations:** 1 Advanced Soft Matter, Department of Chemical Engineering, Faculty of Applied Sciences, Delft University of Technology, Van der Maasweg 9, 2629 HZ Delft, The Netherlands; s.j.picken@tudelft.nl; 2 Environmental Biotechnology, Department of Biotechnology, Faculty of Applied Sciences, Delft University of Technology, Van der Maasweg 9, 2629 HZ Delft, The Netherlands; p.k.wilfert@tudelft.nl

**Keywords:** biopolymers, consistency index, Herschel–Bulkley, intrinsic viscosity, charge screening

## Abstract

The use of the consistency index, as determined from fitting rheological data to the Herschel–Bulkley model, is described such that it may yield systematic trends that allow a very convenient description of the dissipative flow properties of linear and branched (bio)polymers in general, both in molecular and weakly associated supramolecular solutions. The effects of charge-mediated interactions by the systematic variation of the ionic strength and hydrogen bonding by a systematic variation in pH, using levels that are frequently encountered in systems used in practice, is investigated. These effects are then captured using the associated changes in the intrinsic viscosity to highlight the above-mentioned trends, while it also acts as an internal standard to describe the data in a concise form. The trends are successfully captured up to 100 times the polymer coil overlap and 100,000 times the solvent viscosity (or consistency index). These results therefore enable the rapid characterization of biopolymer systems of which the morphology remains unknown and may continue to remain unknown due to the wide-ranging monomer diversity and a lack of regularity in the structure, while the macromolecular coil size may be determined readily.

## 1. Introduction

The prevalent strategy to characterize biopolymers using a range of different spectroscopy techniques is predicated upon the effort to understand their underlying chemical structure. Common examples include the use of Fourier-Transform Infrared spectroscopy (FTIR) to determine the structures of proteins and polysaccharides [1,2,3] and the use of Nuclear Magnetic Resonance (NMR) to determine the structure of polysaccharides in solution [4,5]. Other highly specific examples include the use of Mass Spectrometry (MS), in combination with other preparatory techniques, for proteomics [6,7,8]. In some cases, a combination of these spectroscopic techniques may also be used to understand the chemical structure [9]. The aim of these techniques has been to lay the foundation that establishes the biopolymer’s monomer composition, the functional groups, and to identify similarities or differences compared to other biopolymers. 

Despite their versatility, spectroscopic techniques cannot provide direct information on the properties of a material or clear insights on a material’s structure–property relationships. They only provide considerable insight into the chemical structure of the material. Obviously, an understanding of the chemical structure alone is not sufficient to suitably extract, process, and utilize various biopolymers. Biopolymers are typically extracted and separated as a concentrated system (>10 kg/m^3^, often as high as 100 kg/m^3^) using techniques such as centrifugation, sedimentation, and filtration together with additional downstream purification steps [10,11,12]. It is worth noting that these concentrations are much higher than the (highly) dilute regime where polymer systems are typically studied. Thus, a close understanding of the rheological properties of the system is required. Similarly, the typical applications of biopolymers in the biomedical, food, agriculture, and building industries look to process them into hydrogels [13], foams [14], coatings [15], and composites [16,17]. In these cases, a good understanding of the structure–property relationships, relating to rheology, which can respond to a very wide range of length scales (0.1–100 µm), is generally considered to be relevant as the basis to transform the same concentrated biopolymer system into a variety of materials. In view of the above, there is clearly a need for the actual determination of the rheological properties of complex (bio)polymer-based systems. This can be used directly in the optimization of the extraction, processing, and application of these systems. In addition, rheological information has an extremely high sensitivity to minor interactions that are nearly impossible to find using FT-IR or NMR. For instance, the viscosity of materials can span at least 16 orders of magnitude [18,19]. Thus, subtle changes in the property of materials are immediately apparent. 

Given that the sensitivities and ability of rheometric techniques to provide high resolution information across multiple length scales remains well established [20], a close empirical assessment of the rheological parameters of concentrated biopolymer systems is needed. One such method is the evaluation of a biopolymer’s viscosity as a function of concentration. This may in turn be used to describe the dynamics of a biopolymer system. Certainly, this approach has been used to describe both the dynamics of uncharged synthetic polymers in solution [20] and synthetic polyelectrolytes in solution [21]. However, the attempt to describe the viscosity of biopolymer systems, and thus their associated dynamics over a given concentration range, remains under development [22,23]. Amongst these, Sayko et al. suggest that it is possible to establish a dependence between the viscosity of concentrated biopolymer solutions and the weight-averaged molar mass (in place of concentration) [22]. This is carried out to isolate different relaxation regimes in the resulting curves (Rouse regime v/s entangled regime), whose respective slopes may be explained using prevalent scaling arguments for (bio)polymers in solution. However, it is worth highlighting that the persistence length (and thus the coil size) is strongly influenced by screening effects in the case of biopolymer systems [24]. Thus, the weight-averaged molar mass becomes an improper standard for mapping changes to viscosity. Based on a study conducted by the authors of this paper [24], the intrinsic viscosity may be used in its place as it is possible to capture changes to the coil size in this way. Moreover, the intrinsic viscosity may be used as an internal standard in cases where the complex chemical structure of the biopolymer system remains unestablished. This remains consistent with the second approach highlighted by Pathak et al., where the determination of the exact hydrodynamic conformation of antibody systems in solution remains highly elusive [23]. This method allows for the fitting of power series expansion curves that are similar to the Huggins equation [20]. In general, however, both approaches cover a very limited concentration range and remain specific to a particular subset of biopolymer systems. 

Covering changes to the viscosity to such a limited extent is not particularly useful in describing the viscous properties of a wide variety of biopolymer systems. As such, it is important to cover the adverse shear thinning effects that are prevalent in the working concentration range of these systems [25]. Equally there is a need to cover the effects of “soft” charge-mediated interactions resulting from changes in pH, conductivity, and temperature, as well as changes to the viscosity due to a biopolymer’s chemical structure. Thus, a strategy to characterize concentrated biopolymer systems using the consistency index from the Herschel–Bulkley model (see graphical abstract) and to link the obtained value to the biopolymer’s concentration by changing conditions such as pH, ionic strength, temperature, branching, and molar mass is proposed below. The approach highlighted covers both methods, i.e., power series expansions motivated using the Huggins equation and power laws motivated by scaling arguments. The aim of providing such a framework is to explain the properties of both known biopolymer systems that are frequently used in practice and novel biopolymer systems of which the chemical structure is unknown. Thus, the framework permits the rapid characterization of a wide variety of biopolymer systems for practical scenarios pertaining to their extraction, processing, and application. 

## 2. The Herschel–Bulkley Approach

Upon attempting to determine the viscosity at elevated concentrations, it is observed that concentrated polymer systems seldom behave as Newtonian fluids and often exhibit shear-thinning behavior [25]. It is also frequently found that the solid-like gel structures resulting from the physical interpenetration of coils (in addition to the existing non-covalent interactions within biopolymers) themselves leads to the development of yield stresses and thixotropy [25]. Thus, polymers in the concentrated regime often exhibit pseudoplastic behavior. Conventionally, the viscosity of polymer solutions is determined using zero-shear viscosity measurements [26,27]. However, the solid-like structures of a yield stressing fluid often have elastic contributions at low shear stresses (or at low shear rates). This then limits the ability to experimentally obtain the expected initial plateau in the viscosity of a shear thinning fluid. What is proposed is to characterize polymeric systems over a very wide range of compositions using the Herschel–Bulkley model, which can describe many types of complex systems within the same framework [25]:

(1) $$\sigma =\sigma_{0}+K{\dot{\gamma }}^{n}$$

Here, σ represents the applied or observed shear stress, $\sigma_{0}$ represents the yield stress depicted by the fluid, and $\dot{\gamma }$ represents the observed or applied shear rate, K represents the consistency index, and n represents the power law index. It is worth noting that in the equation above, the units of K are arbitrarily defined based on the value of n. Thus, the Herschel–Bulkley equation may be suitably rewritten to restore dimensional consistency for K (in stress units) irrespective of the value of n:

(2) $$\sigma =\sigma_{0}+K{\left(\frac{\dot{\gamma }}{1\;s^{-1}}\right)}^{n}$$

As the Herschel–Bulkley model by itself holds no theoretical premise, it is worth discussing how it may be interpreted within this study. In cases where the value of the yield stress drops to zero and the value of the power law index is unity, Equation (2) reduces to Newton’s law of viscosity, with the consistency index term representing the viscosity of the Newtonian fluid. In the case of a yield stress and a power law index not equal to 1, the consistency index represents the dissipative contribution to the stress at 1 s^−1^ shear rate. It is thus proposed that the consistency index might be used in place of viscosity to establish global trends in the behavior of biopolymeric systems up to high concentrations. Thus, similar to the Huggins equation [20,23], it is possible to estimate the consistency index of a concentrated biopolymer system using a power series expansion:

(3) $$K=K_{s}\left(1+\left[\eta \right]c+a_{2}{\left(\left[\eta \right]c\right)}^{2}+a_{3}{\left(\left[\eta \right]c\right)}^{3}+\cdots \right)$$

where $K_{s}$ = $\eta_{s}*1\;s^{-1}\;$ is the stress developed by the solvent at $1\;s^{-1}$ shear rate ($\eta_{s}=$ solvent viscosity). This may be suitably rearranged to yield a non-dimensional (universal) equation in terms of the “relative consistency index” (K/$K_{s}$) and “overlap factor” term ($\left[\eta \right]c$) [20,23]:

(4) $$\frac{K}{K_{s}}=1+\left[\eta \right]c+a_{2}{\left(\left[\eta \right]c\right)}^{2}+a_{3}{\left(\left[\eta \right]c\right)}^{3}+\cdots$$

Equally, as is typical from scaling approaches [20], it is possible to represent the relative consistency index as a power law function with respect to the overlap factor (for $\left[\eta \right]c>1)$:

(5) $$\frac{K}{K_{s}}\propto {\left(\left[\eta \right]c\right)}^{\alpha }$$

Note that the relative consistency index is the analog of the relative viscosity, and, indeed, at zero concentration, there is no distinction between $K_{s}$ and $\eta_{s}$ (solvent viscosity). The overlap factor represents the general idea that the change in consistency index should be scaled with respect to the space filling concentration of the macromolecular objects in the system. Even at concentrations up to 100 kg/m^3^, these systems will frequently have a low solid content so that the molecules (or macromolecular objects) can indeed overlap and occupy the same volume. Thus, 1/$\left[\eta \right]$ is interpreted to be the value for the transition from a dilute to a semi-dilute system. 

## 3. Materials and Methods

### 3.1. Materials 

All polymers used in this study were procured from Sigma-Aldrich and are tabulated in Table 1. The authors report [24] that the specific choice of biopolymers offers comparison between linear polyanionic (Na–Alginate), polycationic (chitosan), and polyampholytic (porcine–gelatin) biopolymers. Equally, it is possible to compare biopolymers with differences in their architecture (Na–Alginate, Na–CMC, and pectin–citrus) and the quoted molar mass. Poly-ethylene glycol in particular was selected, for the sake of comparison, by virtue of being a synthetic water-soluble polymer. 

**Figure 1:**
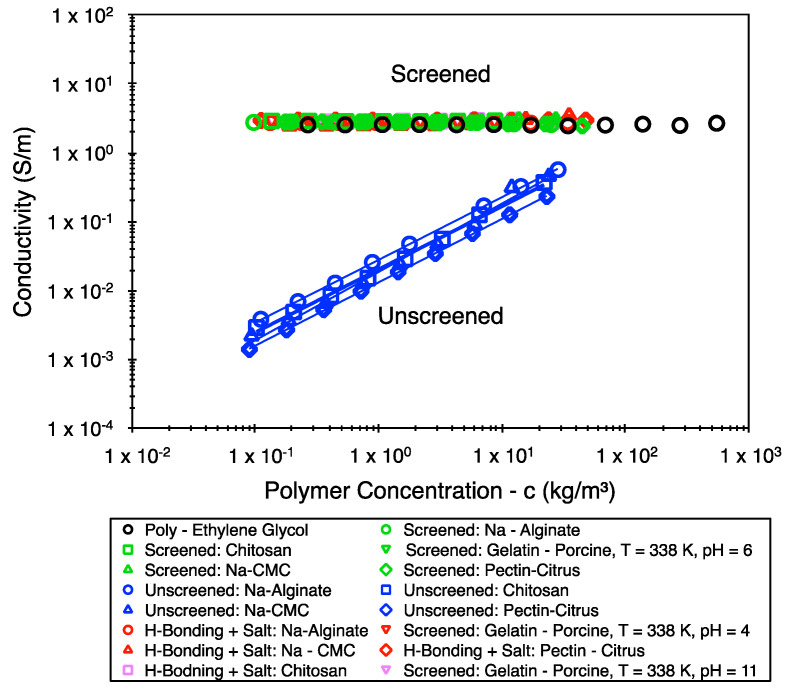
Conductivity of the samples tested (including those reported in [24]). Guides to the eye are also provided for the “Unscreened” samples to highlight the differences in the values of their conductivity and their (somewhat) linear dependence on concentration.

All biopolymer systems were prepared by stirring the desired concentrations in deionized water for 24 h (86,400 s) in a sealed conical flask at 293 K. Increases to the conductivity were achieved by adding 0.2–0.3 M of NaCl to a fraction of the dissolved systems. Equally, hydrogen bonding was introduced by adjusting the pH using 1M HCl and 1M NaOH solutions. The 1M HCl and 1M NaOH solutions were also used to adjust the pH following dilution from higher concentrations. Figure 1 shows the conductivity of the samples (also those reported in [24]—see Supplementary Materials Section S1 for details about the pH of samples). As reported in [24], the polymer concentrations being represented on the x-axis of all figures are believed to be within an error margin of 11% or 0.0453 decades. 

### 3.2. Methods

The consistency index was obtained for the concentrated biopolymer systems (samples not reported in [24]) using a stress-controlled TA Instruments Discovery Hybrid Rheometer–3 (DHR-3). The samples were tested using a steel cone on plate setup with the use of a solvent trap. The smooth steel cone had a diameter of 40 mm and a cone angle of 2°, 0 min, and 50 s (0.035 rad). The truncation gap was maintained at 60 µm over the course of the experiment. All tests were performed at a temperature of 298 K. 

The tests included performing a set of four linearly decreasing stress-controlled flow ramps between prescribed limits over a period of 60 s. The lower limit of these ramps was always prescribed to a zero-stress value. The first ramp was performed as a conditioning ramp to erase the sample history. The subsequent ramps were performed with rest times of 10 s, 100 s, and 1000 s to validate that the protocol yields reproducible results. The average consistency index from the three measurements was obtained along with a standard deviation by fitting the Herschel–Bulkley parameters to the three ramps with intermittent rest times. The fitting was carried out using the scipy.optimize.curve_fit function on Python. The undesired features of the flow ramp curve such as initial inertial effects and the elastic recovery region of the flow ramp at low shear rates were removed before the fitting procedure. The reader is asked to refer to Supplementary Materials Section S2 for more information about the Herschel–Bulkley curve fitting methodology. 

In the case of some samples that presented low consistency index values (typically 10–100 mPa), it could not be ascertained if the cone on plate setup yielded a flow ramp curve with a linear Newtonian slope. This was because such samples produced high shear rates at low stress values, thereby making them difficult to test using the cone on plate setup. In such cases, the concentric cylinder setup was used to obtain a set of data points at five discrete stress values. The test was performed using a steel cup of diameter 30.36 mm and a steel DIN bob rotor with a diameter of 28.00 mm and a height of 42.07 mm. The DIN bob was maintained at a height of 5917.1 µm from the bottom surface of the cup. All tests were performed at a temperature of 298 K. The Herschel–Bulkley parameters were again fitted to these five data points to obtain the consistency index value along with a standard deviation. 

## 4. Results

**Figure 2:**
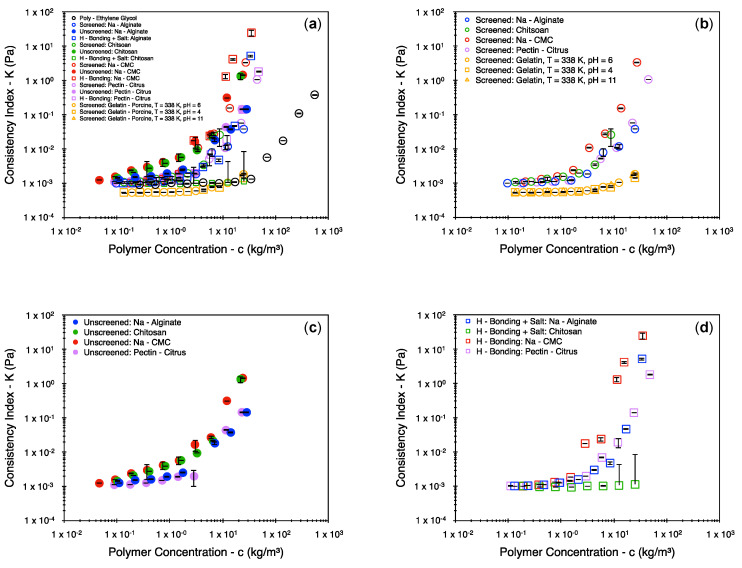
Consistency index as a function of polymer concentration (**a**) for all biopolymer systems, (**b**) for unscreened biopolymer systems, (**c**) for screened biopolymer systems, and (**d**) for hydrogen-bonded systems. The vertical error bars represent the two-sigma distribution (95% confidence interval).

**Figure 3:**
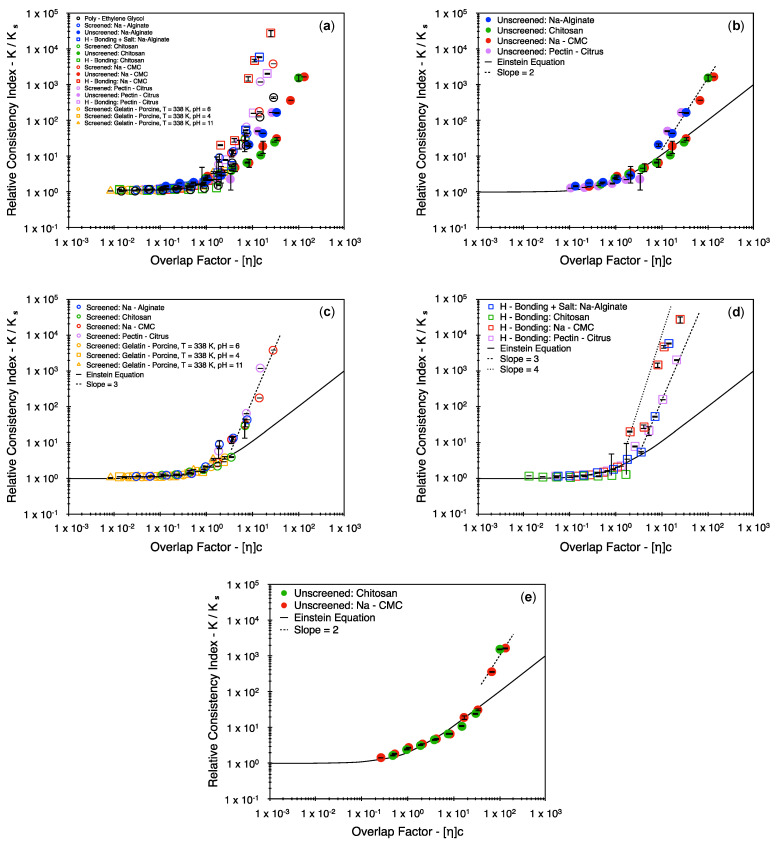
The relative consistency index v/s the overlap factor (**a**) for all systems, (**b**) for unscreened systems, (**c**) for screened systems, (**d**) for hydrogen-bonded systems, and (**e**) for the unscreened chitosan and Na–CMC systems to highlight their delayed departure from the Einstein equation $(\left[\eta \right]c$ > 10). The vertical error bars represent the two–sigma distribution (95% confidence interval).

Figure 2 represents the obtained consistency index plotted against the polymer concentration. The vertical error bars are indicative of the two-sigma (95%) confidence interval. This interval is small in most cases. The cases that present with the largest errors are the ones evaluated at discrete stress values using the concentric cylinder setup. Thus, the error in estimating the consistency index with a greater precision is attributed to the fitting of the nonlinear Herschel–Bulkley model to a limited set of data points. A deviation from a linear set of trends is presented by almost all data sets of Figure 2, with the only exception being chitosan at elevated pH conditions with added salts (green unfilled squares). However, the solvent’s viscosity is also impacted by the salinity and temperature at which the system was tested, thereby shifting data sets vertically. Similarly, all data sets are shifted horizontally by virtue of changes to the intrinsic viscosity. To account for this, it is important to represent the results of Figure 2 in terms of the relative consistency index and the overlap factor. The rescaled data sets are presented in Figure 3. The rescaling was possible due to the intrinsic viscosity calculations that were made at a low polymer concentration. Most of the intrinsic viscosity values are presented in [24]. However, to aid interpretability, the entire list of values is report in Table 2 (conversion to the frequently used dL/g may be obtained upon multiplying all values by 10). 

As anticipated, the data sets start showing deviations from the Einstein equation around the overlap factor value of $\left[\eta \right]c$ = 1, except for the unscreened CMC and chitosan systems (green filled circles and red filled circles, respectively), where deviations were observed when $\left[\eta \right]c$ > 10 (See Figure 3e). Also, when presented in this fashion, the underlying similarities in the data sets become evident. To begin with, it is easy to describe the underlying differences in the various biopolymer systems in their deprotonated state (circles–filled and unfilled). Irrespective of the type of polyelectrolyte under consideration (polyanions or polycations), it appears that once the charged interactions are screened by increasing the ionic strength in the system, the relative consistency index may be described using a third-order power series expansion, or a power law curve with an α value around 3 (2.6 to 3.4—Figure 3b). However, when the charged interactions remain unscreened, the relative consistency index may be described using a second-order power series expansion, or a power law curve with an α value around 2 (1.7 to 2.3—Figure 3c). 

In cases where hydrogen bonding was introduced, the third-order trends continue to persist in the case of the polyanions (Figure 3d), with the change in the degree of association being reflected only in the value of the intrinsic viscosity. In this case, α values from 3 to 4 offer reasonable power law fits. However, a consistent rise or fall is not reflected in the value of intrinsic viscosity. In the case of alginate and pectin, this value rises roughly by a factor of 2; however, in the case of CMC, this value falls roughly by a factor of 1.4 (Figure 3c,d, Table 2). Expectedly, the introduction of associative interactions within the coils and their associated changes in the coil dynamics are exclusive to the polymer system under consideration. This point is further highlighted upon comparing the results for chitosan with the results of the polyanions. When the pH is raised for chitosan, the intrinsic viscosity value drops roughly by a factor of 6.3 to suggest a strong association within the coils.

## 5. Discussion

### 5.1. Exploring the Theoretical Premise

Upon examining the trends in Figure 3, it is noticeable that they follow from a logical interpretation of screening charged interactions. When the charges on a polymer chain remain unscreened and when $\left[\eta \right]c>1$, a charged polymer chain is likely to experience repulsions from neighboring like charges by virtue of a drop in the correlation length. Thus, the overlapping coils are likely to take up a collapsed conformation to mitigate interactions with neighboring chains. In some cases, such as CMC and chitosan, these repulsive interactions may be expected to dominate up to $\left[\eta \right]c\approx 10$, thereby leading to a delayed onset for the deviation from the Einstein equation. However, once the polymer–polymer interactions are screened with the addition of salts, the charged sites of the coils are freer to overlap, leading to a sharper increase in the viscous dissipation term (the consistency index). 

This is analogous to solvent quality-mediated interactions in uncharged polymers and their influence on the second-order virial coefficient term [20]. In the case of synthetic polymers that are in a good (athermal) solvent, the excluded volume interactions are expected to dominate, thereby leading to a large second-order virial coefficient term. However, as the system approaches the theta condition, the excluded volume approaches zero, thereby plausibly leading to a domination of the three-body interaction term at higher concentrations (the third-order coefficient). Under theta conditions, the polymer–polymer interactions are imagined to be negligible (the second-order coefficient). Pathak et al. [23] also report a more in-depth analysis of the Huggins coefficient term and its potentially predictable correlation to the second virial coefficient term. However, it is reported that a direct correlation could not be found to link the terms together. In part, this shortcoming was also attributed to the failure of existing physical models for polymers. In contrast, Sayko et al. [22] managed to link the excluded volume interaction term(s) and changes to the ionic strength of the system and suggested that screening can indeed be used to minimize the excluded volume interactions. 

**Figure 4:**
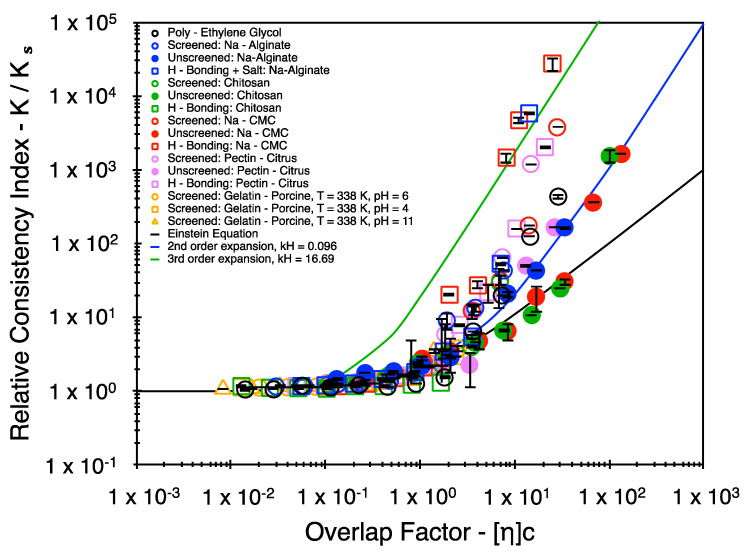
Trends in the relative consistency index explained using the Huggins equation. Note that the data points are carried over from Figure 3.

In a similar light, previous literature sources suggest that the power series expansion may be limited to include only a second-order correction factor at elevated polymer concentrations and thus recommend to only use the Huggins coefficient term ($k_{H}$) as a descriptive term to describe the polymer–solvent and polymer–polymer interactions [28,29]. Among these, Lewandowska et al. suggest comparatively lower $k_{H}$ values for polymers in good solvents and higher $k_{H}$ values for polymers in theta solvents or polymer coils that are associated via hydrogen bonding. Upon making a similar attempt to restrict the fit to only a second-order power series expansion, a similar increase in the value of $k_{H}$ is noticeable. However, large deviations are observed between the fit and the experimental data (Figure 4). This clearly suggests the necessity for (at least) a third-order term in the power series expansion to account for the contribution of the plausible three body interactions. 

Unlike the power series expansions, a strong theoretical justification is not provided here for the found power law indices. As suggested in some sources [26,27], the precise values of the power law indices are highly specific to the type of network structure and the strength of the entangled junction points at elevated concentrations, as these would dictate the ease of reptation and the time scales for relaxation. Indeed in the case of the PEG system, it is found that the results may be suitably represented using a power index between 2 and 3. Given that the underlying differences in the chemical structure and branching between different polymer systems is clearly identifiable, the choice of power law indices may not be appropriate for all systems described here, but only for linear systems that are best described by a fractional power law dependence curve. It is worth highlighting, however, that these may not be easily arrived at for all systems, as the fractional power law index has been further developed only relatively recently for linear systems [30]. 

Thus, it is recognizable that there are still some theoretical objections to both approaches, i.e., power series expansions and scaling arguments. However, by trying to adopt both approaches, an attempt is made to highlight that there is a consistent set of experimentally observable trends that are still highly relevant for the extraction, processing, and utilization of biopolymers in practical situations. At the very least, the approach of using the overlap factor term has also facilitated the explicit highlighting of the changes to the consistency index value due to screening effects in concentrated systems. Indeed, the hope is that, in due course, further clarity might be obtained by analyzing a wide range of polymeric materials using the approach described here. 

### 5.2. Yield Stress and Power Law Indices

**Figure 5:**
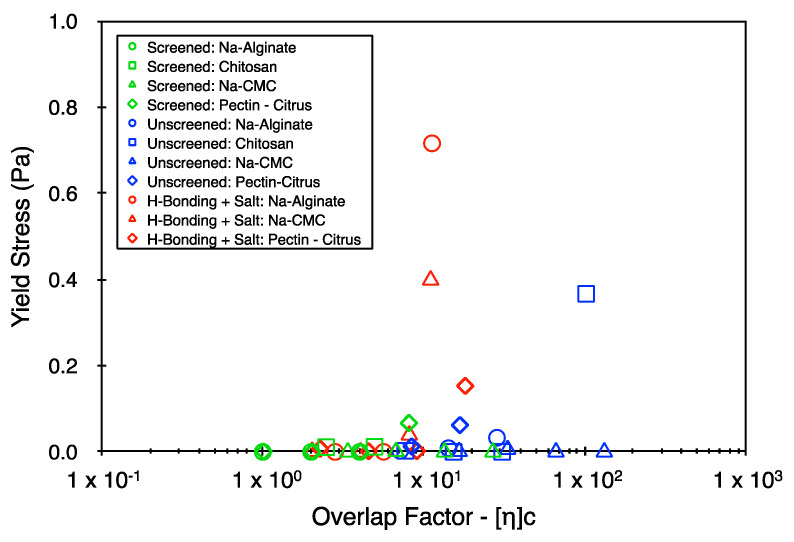
Yield stress from the Herschel–Bulkley fitting as a function of the overlap factor.

**Figure 6:**
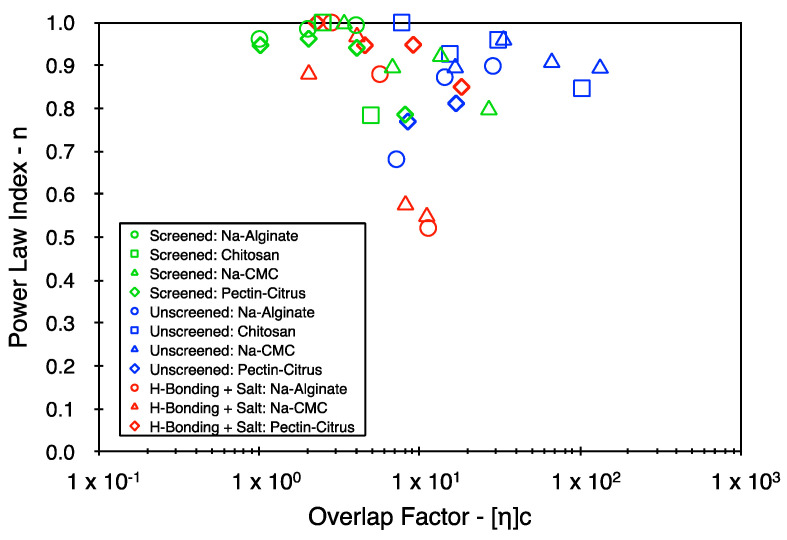
Power indices from the Herschel–Bulkley fitting as a function of the overlap factor.

The success in elucidating trends using the consistency index suggests that the approach may be extended to include the other two Herschel–Bulkley parameters. Figure 5 and Figure 6 represent the yield stress and the power law index values, respectively, for a select set of data points from the Herschel–Bulkley fitting. It is clear from Figure 5 that there is no consistent set of trends in the yield stress with respect to the concentration, with most samples exhibiting large variations in its value. Upon comparing the results of Figure 5 with those of Figure 6, the samples that present the largest yield stress values also typically present the lowest flow indices, suggesting a high degree of physical interaction between the polymer coils. However, the origin of these interactions is distinct in different systems. For example, in the case of the hydrogen-bonded alginate samples (red circles), the system enters an associated state with its macro-state being a granular fluid with potential noncovalent interactions between the soft (easily deformable) suspended particles. In contrast, the physical interactions in the unscreened and deprotonated alginate samples (blue circles) potentially correspond only to the entanglement of polymer coils (at relatively high concentrations compared to the hydrogen-bonded systems), with the bulk fluid being macromolecularly dissolved. Thus, the yield stress appears to be much more sensitive to an entire range of dynamic physical interactions that can persist within the concentrated biopolymer systems [31,32]. The approach taken here is to explore the analogies (if any) between the dissipative part of the flow behavior of a wide range of polymer systems. However, the yield stress also presents itself as an important physical property of concentrated biopolymer systems and deserves rigorous assessment separately.

## 6. Conclusions

A framework is provided to characterize biopolymer systems up to 100 times the overlap concentration and up to 100,000 times the solvent viscosity, using a set of experimentally observable trends for the Herschel–Bulkley consistency index. The range of values expressed here are significantly larger than previously reported values and should cover concentrations typically encountered in practice. In some cases, the observed trends agree with what, from theoretical arguments, should be found for uncharged, synthetic–linear polymer chains. The analysis is performed using either a generalized power series expansion (inspired by the Huggins equation) or a power law (scaling arguments). However, the proposed method also works for polymer systems that involve branching and exhibit dynamic crosslinking and the coil-to-rod transition type of phenomena (related to screening and solvent quality). Thus, there is sufficient scope to develop the theoretical arguments in support of the observed trends for complex biopolymer systems, as these may be used further to highlight the dynamics of biopolymer chains in solution. Regardless, this method provides the rapid determination of universal experimental trends, particularly at higher concentrations, and is highly useful for the efficient formulation of dedicated biopolymer products, as they invariably rely on good control over the rheology.

## Figures and Tables

**Table 1:** List of polymers and their salient properties: type of functionalization, architecture, and molar mass ^1^.

Polymer Name	Salient Properties
Poly-Ethylene Glycol (PEG)	End functionalization with hydroxyl group, $\mathrm{Avg}.\;M_{n}\;$= 20 kg/mol.
Sodium Alginate (Na–Alginate)	Polyanion: Carboxyl group, High mannuronic acid content. Linear copolymer.
Chitosan	Polycation: Amine group, Linear homopolymer, $M_{w}$~50 to 190 kg/mol.
Sodium Carboxy Methyl Cellulose (Na–CMC)	Polyanion: Carboxyl group, Degree of substitution = 0.9, Linearly substituted homopolymer, $M_{w}$~250 kg/mol.
Pectin from Citrus Peels (Pectin–Citrus)	Polyanion: Carboxyl group, Galacturonic acid ≥ 74.0%, degree of methylation ≥ 6.7%, Branched heteropolymer.
Gelatin from Porcine Skin (Porcine–Gelatin)	Polyampholyte: Carboxyl group and amine group, Linear–collagen derivative.

^1^ Molar mass (or range) is reported wherever it is provided by manufacturer.

**Table 2:** Experimentally obtained values for the intrinsic viscosity.

Biopolymer System	[η] (m^3^/kg) ± S.D. ^1^
Poly–Ethylene Glycol	0.053 ± 0.007
Screened: Na-Alginate	0.308 ± 0.065
Screened: Chitosan	0.795 ± 0.100
Screened: Gelatin–Porcine, T = 338 K, pH = 6	0.102 ± 0.024
Screened: Na-CMC	1.034 ± 0.037
Screened: Pectin–Citrus	0.332 ± 0.064
Unscreened: Na-Alginate	1.183 ± 0.157
Unscreened: Chitosan	4.636 ± 0.379
Unscreened: Na-CMC	5.624 ± 0.626
Unscreened: Pectin–Citrus	1.152 ± 0.175
H-Bonding + Salt: Na–Alginate	0.420 ± 0.042
Screened: Gelatin–Porcine, T = 338 K, pH = 4	0.095 ± 0.017
H-Bonding + Salt: Na-CMC	0.719 ± 0.016
H-Bonding + Salt: Pectin–Citrus	0.440 ± 0.027
H-Bonding + Salt: Chitosan	0.066 ± 0.027
Screened: Gelatin–Porcine, T = 338 K, pH = 11	0.060 ± 0.017

^1^ S.D. = standard deviation.

## Data Availability

The original data presented in this study are openly available in the 4TU.ResearchData repository at https://doi.org/10.4121/f1ab8cce-67ec-4a8d-8f3b-4471db5d372e.

## Supplementary Materials

The following supporting information can be downloaded at https://www.mdpi.com/article/10.3390/polym16192822/s1, Section S1: pH of Samples; Section S2: Fitting the Herschel–Bulkley Model.
