# Supplementary material for: Using the Herschel–Bulkley Consistency Index to Characterise Complex Biopolymer Systems—The Effect of Screening
Source: Polymers (Basel). 2024 Oct 6;16(19):2822. doi: 10.3390/polym16192822 (PMC11478448; doi:10.3390/polym16192822)
Supplement: Supplementary file 1 [file polymers-16-02822-s001.zip › polymers-3244860-supplementary.pdf]

Supplementary Material for Publication

Using the Herschel–Bulkley Consistency Index to Characterise Complex Biopolymer Systems—The Effect of Screening

Anand Raja [1,*], Philipp K. Wilfert [2,†] and Stephen J. Picken [1]

[1] Advanced Soft Matter, Department of Chemical Engineering, Faculty of Applied Sciences, Delft University of Technology, Van der Maasweg 9, 2629 HZ Delft, The Netherlands; s.j.picken@tudelft.nl

[2] Environmental Biotechnology, Department of Biotechnology, Faculty of Applied Sciences, Delft University of Technology, Van der Maasweg 9, 2629 HZ Delft, The Netherlands; p.k.wilfert@tudelft.nl

* Correspondence: a.raja-1@tudelft.nl; Tel.: +31-622802977

† Present Address: Urban Water Management, University of Applied Sciences—Lübeck, Mönkhofer Weg 239, 23562 Lübeck, Germany.

S1. pH of Samples:

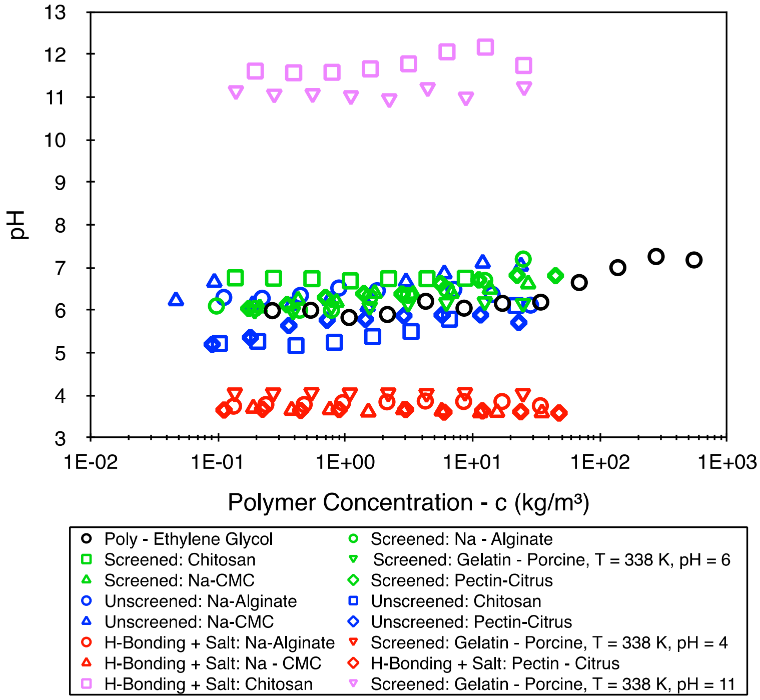

Figure S1: This figure shows the pH of all the samples that were tested.

S2. Fitting the Herschel-Bulkley Model

When performing a linear flow ramp experiment, it is possible to carry out both, increasing flow ramps (ramp up) as well as decreasing flow ramps (ramp down). However, the ramp down was particularly chosen to overcome the stress overshoot that typically results upon start-up flows in complex fluids (Figure S2). This also guaranteed clearly distinguishable features such as the yield stress (intercept made with the y-axis) whilst preserving the shear thinning behavior (shape of the curve).

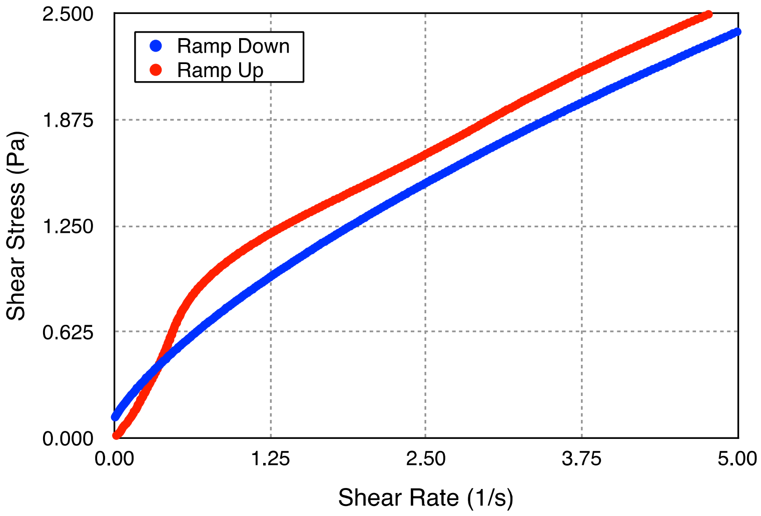

Figure S2: This figure shows the linear flow ramp curves performed for the same sodium alginate sample (pH = 3.72, polymer concentration = 2.87 g/dL, no added salts).

As mentioned in the main text, multiple ramps were performed with intermediate rest times of 10, 100 and 1000 seconds to validate that our protocol yields reproducibility in the results to a great extent. Figure S3 further highlights this point and additionally confirms that the uncertainty associated with fitting the Herschel-Bulkley parameters is negligible.

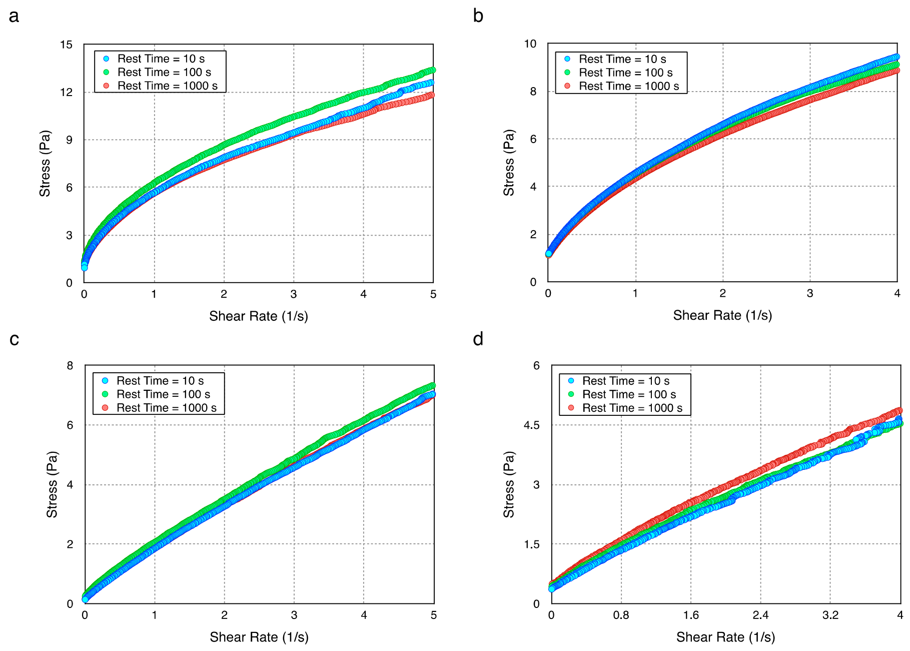

Figure S3: This figure shows the linear flow ramp down curves performed at different rest times for the following biopolymer systems: (a) sodium alginate - low pH + salt, polymer conc. = 3.41 g/dL; (b) sodium carboxy methyl cellulose - low pH + salt, polymer conc. = 1.56 g/dL; (c) citrus pectin - low pH + salt, polymer conc. = 4.78 g/dL; and (d) chitosan – neutral pH + no salt, polymer conc. = 2.19 g/dL.

Given the non-linear nature of the Herschel-Bulkley model, the scipy.optimise.curve_fit() function from the SciPy library (Python programming language) was used. This function makes use of a non-linear least squares method to fit the chosen model to the data. Additionally, the function also provides the ability to conveniently change the initial guesses for our parameters of interest, the bounds of these parameters and returns the covariance matrix (Figure S4). As the parameters of interest in the Herschel-Bulkley model are the yield stress, the consistency index and the power law index, the resulting covariance matrix is a 3x3 matrix. The first row and column correspond to the yield stress covariance terms, the second row and column correspond to the consistency index covariance terms and the third row and column correspond to the power law index covariance terms respectively. The scipy.optimise.curve_fit() was also used subsequently to fit the intrinsic viscosity values and the coefficient values for the power series expansions. Additional documentation about the function and its implementation within a Python environment is available on the following webpage: https://docs.scipy.org/doc/scipy/reference/generated/scipy.optimize.curve_fit.html .

```
[[ 7.22524313e-07 -3.98967735e-07  2.80410873e-05]
 [-3.98967735e-07  2.62383645e-07 -1.92955121e-05]
 [ 2.80410873e-05 -1.92955121e-05  1.44253646e-03]]
```

Figure S4: This figure represents the typical covariance matrix that is obtained using the scipy.optimise.curve_fit() function. The low values represented here are indicative of the high-quality fits that are possible using this method. The parameters are respectively: yield stress, consistency index, and the power law index.
